# Disparities in food insecurity between sexual minority and heterosexual adults – a higher burden on bisexual individuals

**DOI:** 10.3389/fpubh.2023.1237091

**Published:** 2023-08-07

**Authors:** Nasser Sharareh, Sara Bybee, Evan Goldstein, Shannon Jones, Rachel Hess, Andrea Wallace, Hilary Seligman, Fernando A. Wilson

**Affiliations:** ^1^Department of Population Health Sciences, Spence Fox Eccles School of Medicine, University of Utah, Salt Lake City, UT, United States; ^2^College of Nursing, University of Utah, Salt Lake City, UT, United States; ^3^Department of Nutrition and Integrative Physiology, University of Utah, Salt Lake City, UT, United States; ^4^Department of Medicine, School of Medicine, University of California, San Francisco, San Francisco, CA, United States; ^5^Matheson Center for Health Care Studies, University of Utah, Salt Lake City, UT, United States; ^6^Department of Economics, College of Social and Behavioral Science, University of Utah, Salt Lake City, UT, United States

**Keywords:** structural racism, discrimination, food insecurity, LGBTQ, health policy, public health

## Abstract

**Background:**

Sexual minorities—individuals who identify as gay/lesbian, bisexual, or other non-heterosexual individuals—experience higher rates of food insecurity (FI) compared to heterosexual individuals. During the COVID-19 pandemic, discrimination and structural racism, which are known risk factors for food insecurity, were perpetuated against sexual and racial/ethnic minorities. However, to our knowledge, a nationally representative analysis of the impact of the pandemic on food insecurity by sexual minority status and based on race/ethnicity is missing. We aimed to determine the degree of association between FI and sexual minority adults overall, before (2019) and during (2020–2021) the pandemic, and stratified by race/ethnicity.

**Methods:**

We used nationally representative data from the 2019–2021 National Health Interview Survey (NHIS). We specified multivariable logistic regression models to determine the association between FI and identifying as a sexual minority adult (≥18 years old), including gay/lesbian, bisexual, and other non-heterosexual individuals.

**Results:**

Overall, we only observed FI disparities between bisexuals and heterosexuals (aOR 1.61 [95% CI 1.31–1.99]). Stratified by year, this association was significant only during the pandemic. Stratified by race/ethnicity, non-Hispanic white and non-Hispanic black individuals identifying as bisexual also experienced a significantly higher FI rate than their heterosexual counterparts.

**Conclusion:**

Our results may be a manifestation of the disproportionate impact of discrimination on bisexual individuals’ FI experiences. With the growing number of legislative bills targeting the rights of sexual minorities, we expect to see a higher burden of FI among bisexuals, particularly, bisexual people of color. Future intersectional research regarding FI among bisexual and racial/ethnic minority individuals would further elucidate how membership in multiple minority groups may contribute to a higher risk of FI.

## 1. Introduction

Food insecurity (FI) is defined by the U.S. Department of Agriculture as the lack of access to adequate food for an active, healthy life (1). Sexual minorities—individuals who identify as gay/lesbian, bisexual, or other non-heterosexual individuals—are at higher risk of FI compared to their heterosexual peers (2–8), due to multiple, often intersecting factors. Sexual minorities are repeatedly the target of discrimination and stigma both in their households and social environments (3, 7, 8). They also tend to have higher rates of poverty (9) and less access to employment opportunities, social support, and healthcare (4, 10). All of these factors can increase FI as well (1, 4, 7–9, 11). In addition, FI is associated with hypertension, obesity, mental illness, and cardiovascular diseases, which are disproportionately experienced by sexual and gender minorities (2). Given 2021 estimates that over 20 million people in the U.S. identified as lesbian, gay, bisexual, transgender, or other sexual or gender minorities (e.g., asexual, pansexual) (12), addressing FI among sexual minorities has the potential to make a significant positive public health impact in the U.S.

FI disparities among sexual minorities may have worsened due to the COVID-19 pandemic. In 2021, sexual minorities were more likely than heterosexual individuals to report economic hardship (i.e., loss of income) (13, 14). Racism and discrimination against racial/ethnic and sexual minorities were also perpetuated during the pandemic through verbal harassment, exclusion from events/activities, violence against Black Lives Matter movements, and discriminatory policies (15–18). However, the strength of the associations between economic hardship and discrimination on FI may vary depending on different sexual minority subgroups (e.g., gay/lesbian versus bisexual individuals). Bisexual individuals in general face higher rates of poverty, discrimination, violence, and stigma compared to gay/lesbian individuals (2, 9, 19–22). A recent analysis of Household Pulse Survey data shows that bisexual individuals reported higher rates of food insufficiency than other sexual minorities (23). In addition, people of color who identified as sexual minorities experienced higher rates of food insufficiency than white heterosexuals, white sexual minorities, and heterosexual individuals of color. Although food insufficiency is a simpler measure than FI and only captures whether people have enough food to eat (23), these results indicate the importance of investigating FI using an intersectional approach that considers membership in multiple historically-minoritized groups such as sexual and racial/ethnic minorities (24, 25).

While several studies have explored FI among sexual minorities using a nationally representative sample (3–7), the most recent analysis was based on data gathered in 2018 (6). Furthermore, the Household Pulse Survey measures food insufficiency not FI and lacks pre-pandemic data for comparison. Therefore, there remains a critical gap in nationally representative research on the impact of the pandemic on FI experiences by sexual minority status and based on race/ethnicity. The purpose of this study was to estimate the association between FI and sexual orientation overall, before (2019) and during (2020–2021) the COVID-19 pandemic, and stratified by race/ethnicity. We hypothesized that sexual minorities, specifically bisexual individuals, experienced a higher burden of FI compared to heterosexual individuals during the pandemic, and sexual minorities who identified as people of color were more likely to report FI compared to heterosexual people of color.

## 2. Methods

This study was a cross-sectional analysis of deidentified, publicly-available data; hence, approval from Institutional Review Board was not required. All the analyses were conducted in March 2023.

### 2.1. Population

We used 3 cycles of the National Health Interview Survey (NHIS) data—2019–2021. NHIS is an in-person health survey of the noninstitutionalized U.S. population conducted by the National Center for Health Statistics (26). In some instances, they call respondents to complete the surveys. During 2020 and because of the COVID-19 pandemic, most of the interviews were conducted through telephone calls. Even considering that, NHIS provides detailed nationally representative data spanning the pandemic, unlike the National Health and Nutrition Examination Survey (27), which has been used in prior research on sexual orientation and FI (4–6). Information about sampling structure and weighting is described elsewhere (26). We restricted our analysis to adults ≥18 years old because NHIS does not pose the sexual orientation question to children (6). We also did not pool data prior to 2019 because NHIS redesigned its content, structure, and questionnaire in 2019, which could result in different estimates for pre-2019 compared with 2019 (26).

### 2.2. Study variables

Dependent variable: A binary variable indicating the experiences of FI in the past 30 days was used as the dependent variable. In NHIS, FI is measured with the USDA’s 10-item Household Food Security Survey Module. This module assesses the household’s financial access to food over the past 30 days. Response options to these 10 questions are based on a 3-category Likert scale: 1 (often true), 2 (sometimes true), and 3 (never true). Affirmative responses (i.e., often or sometimes true) are summed to determine food security status, with 3 or more affirmative responses considered food insecure. This includes the USDA categories of low food secure (in which participants report 3–5 affirmative responses), and very low food secure (6 or more affirmative responses).

Primary Independent Variable: The main independent variable of interest was self-reported sexual orientation. Sexual orientation is based on a person’s sexual identity, attraction, and behavior (28, 29). NHIS measures sexual orientation by sexual identity only, which cannot capture gender minorities (29). Sexual orientation was determined by asking adults the following question: “Do you think of yourself as gay/lesbian; straight (that is not gay/lesbian); bisexual; something else; or you do not know the answer?” A categorical variable was used to indicate whether a person is heterosexual, gay/lesbian, bisexual, or other non-heterosexual individuals (i.e., something else), which could include identities such as asexual and pansexual. Individuals were excluded if they refused to answer, or their response was “I do not know the answer.”

Other independent variables: Participants’ race/ethnicity was collected by asking adults the following questions: “Do you consider yourself Hispanic/Latino?” and “What race do you consider yourself to be?” Participants’ race/ethnicity were categorized as non-Hispanic white, non-Hispanic black, Hispanic, and non-Hispanic other. We also adjusted our analysis for variables that are known to be associated with FI such as immigration status, income-poverty ratio, Supplemental Nutrition Assistance Program (SNAP) utilization, smoking status, and disability (3–6, 11). See Appendix A in the Supplement for the full list of independent variables and the NHIS question used to assess each variable.

### 2.3. Statistical analysis

All analyses were conducted in R Statistical Software [v4.1.1; R Core Team (30)] using the survey package and by accounting for complex survey design. R codes are provided in Appendix B in the Supplement. We used descriptive statistics to describe independent variables for the full sample and stratified based on sexual orientation. We then fitted a multivariable logistic regression model to the complete cases (i.e., without missing data) to determine key determinants of FI disparities among sexual minorities. Using subpopulation analysis, we also stratified our analysis based on year and race/ethnicity (i.e., assigning a zero weight to survey respondents outside of the subgroup of interest). When less than 10% of data is missing, ignoring cases with missing information poses minimal bias on results (31), which was the case for both of our multivariable and stratification analyses. We calculated the adjusted odds ratios (aOR) and their 95% confidence intervals (CI) for all models. We also evaluated the multicollinearity among independent variables for each model and found that none of them were highly correlated.

## 3. Results

A total of 93,047 adults were interviewed by NHIS; 30,115 adults in 2019, 29,533 adults in 2020, and 27,454 adults in 2021 (see Table 1). Among interviewed adults, 85,913 (95.94%) identified as heterosexual, 1,625 (1.72%) as gay/lesbian, 1,396 (1.83%) as bisexual, and 441 (0.50%) as other non-heterosexual individuals. The remaining 3,672 adults either refused to answer or did not know the answer and hence were excluded from our analysis. Overall, 7.38% of respondents reported living in a food-insecure household in the past 30 days. Bisexual individuals and other non-heterosexual individuals reported a higher rate of FI compared to heterosexual and gay/lesbian individuals; 16.98 and 13.28%, respectively.

**Table 1:** Descriptive statistics of the total sample and by sexual orientation.

Variable	All (*n* = 93,047) weighted %	Heterosexual (*n* = 85,913) weighted %	Gay/Lesbian (*n* = 1,625) weighted %	Bisexual (*n* = 1,396) weighted %	Other non-heterosexual (*n* = 441) weighted %
Food security status					
Food secure	92.62	92.90	92.33	83.02	87.72
Food insecure	7.38	7.10	7.67	16.98	13.28
Sexual orientation					
Heterosexual	95.94	NA	NA	NA	NA
Gay/Lesbian	1.72	NA	NA	NA	NA
Bisexual	1.83	NA	NA	NA	NA
Other non-heterosexual	0.50	NA	NA	NA	NA
Immigration status					
U.S. Born	81.82	81.47	90.85	93.68	88.97
Non-citizen	8.08	8.24	3.48	2.82	5.57
Naturalized citizen	10.10	10.29	5.66	3.49	5.46
Race/Ethnicity					
Non-Hispanic white	62.97	63.35	65.36	65.69	62.87
Non-Hispanic black	11.71	11.50	13.19	9.36	11.58
Hispanic	16.74	16.67	13.73	16.22	13.68
Non-Hispanic other	8.59	8.48	7.71	8.72	11.87
Marital status					
Not married	47.99	46.52	73.15	78.68	76.67
Married	52.00	53.48	26.85	21.32	23.32
Sex					
Male	48.29	48.81	56.91	24.77	36.16
Female	51.70	51.19	43.09	75.23	63.84
Age					
18–37	29.34	28.12	42.22	70.50	54.38
35–49	24.16	24.32	23.63	18.69	20.78
50–64	24.70	25.21	23.74	7.33	13.06
65+	21.80	22.35	10.41	3.48	11.78
Education					
No diploma	5.51	5.46	1.39	3.23	4.06
GED or high school	19.74	19.72	13.85	16.67	13.44
Some college	30.25	30.12	30.57	39.27	34.77
Bachelor	25.42	25.46	28.90	26.40	28.98
Higher graduate	19.08	19.22	25.29	14.42	18.74
SNAP utilization					
Not a SNAP utilizer	88.11	88.39	87.72	80.06	84.97
SNAP utilizer	11.89	11.61	12.28	19.94	15.03
Self-reported health status					
Excellent or very good	58.22	58.63	65.46	52.37	51.36
Good	27.55	27.45	23.81	32.80	29.97
Fair or poor	14.23	13.92	10.73	14.83	18.66
Smoking status					
Not a current smoker	87.34	87.50	54.94	81.00	85.77
Current smoker	12.66	12.50	15.06	18.00	14.23
Having difficulties paying for medical bills?					
No difficulties	87.88	88.16	88.09	78.61	84.84
Yes	12.12	11.84	11.91	21.39	15.16
Having difficulties paying for medications					
No difficulties	94.21	94.46	92.60	85.95	87.13
Yes	5.78	5.53	7.40	14.05	12.87
Income-poverty ratio					
>200 FPL	71.52	71.97	77.80	61.61	67.15
<100 FPL	10.36	10.07	8.62	16.23	12.59
100–200 FPL	18.11	17.95	13.56	22.16	20.25
Having health insurance					
No health insurance	8.95	8.79	6.94	13.10	10.79
With health insurance	91.05	91.20	93.06	86.90	89.21
Household size					
1 Member	15.70	15.38	23.40	15.76	20.51
2 Members	34.44	34.48	42.22	32.88	40.44
3 Members	25.56	25.44	23.76	34.89	26.64
> = 4 Members	24.30	24.70	10.62	16.48	14.40
Disability					
Living without a disability	91.13	91.36	92.93	88.92	88.30
Living with a disability	8.87	8.64	7.07	11.08	11.70
Region					
West	23.66	23.46	28.04	26.10	29.78
Northeast	17.61	17.73	20.46	15.58	19.52
Midwest	20.90	21.12	14.83	24.82	21.18
South	37.83	37.98	36.67	33.51	29.51
Metropolitan status					
Metro area	86.07	85.73	92.69	89.18	89.72
Non-metro area	13.93	14.27	7.31	10.82	10.28
Year					
2019	33.18	33.55	25.78	26.31	27.43
2020	33.33	33.52	34.18	30.68	32.27
2021	33.48	32.93	40.05	43.01	40.29

General Educational Development (GED), Supplemental Nutrition Assistance Program (SNAP), Federal Poverty Level (FPL).

### 3.1. Multivariable regression model

Table 2 shows the results of the multivariable model adjusted for all the independent variables in Table 1. Even after adjusting for variables such as income-poverty ratio, SNAP utilization, race/ethnicity, health status, insurance, and difficulties paying for medical bills and medications, we observed significant FI disparities between bisexual and heterosexual individuals (aOR 1.61 [95% CI 1.31–1.99]) but not between gay/lesbian or other non-heterosexual individuals and heterosexual individuals. The highest aOR was for those with an income below 100% federal poverty level (FPL) (aOR 4.25 [95% CI 3.75–4.83]).

**Table 2:** Food insecurity and its association with sexual orientation after multivariable logistic regression adjustment.

Variable	Adjusted odds ratio (95% confidence interval)
Sexual orientation (Ref: Heterosexual)	
Gay/Lesbian	1.07 (0.80–1.42)
Bisexual	1.61 (1.31–1.99)***
Other non-heterosexual	1.53 (0.96–2.44)
Immigration status (Ref: U.S. Born)	
Non-citizen	1.38 (1.17–1.62)***
Naturalized citizen	1.21 (1.04–1.41)***
Race/Ethnicity (Ref: Non-Hispanic white)	
Non-Hispanic black	1.80 (1.60–2.01)***
Hispanic	1.30 (1.14–1.47)***
Non-Hispanic other	1.42 (1.17–1.73)***
Married (Ref: Not married)	0.82 (0.74–0.90)***
Female (Ref: Male)	1.07 (0.99–1.16)
Age (Ref: 18–34)	
35–49	1.03 (0.92–1.15)
50–64	0.84 (0.74–0.95)**
65+	0.49 (0.43–0.56)***
Education (Ref: No diploma)	
GED or high school	0.93 (0.81–1.07)
Some college	0.92 (0.80–1.06)
Bachelor	0.58 (0.49–0.69)***
Higher graduate	0.40 (0.33–0.50)***
SNAP utilizer (Ref: Not using SNAP)	2.11 (1.90–2.34)***
Self-reported health status (Ref: Excellent or very good)	
Good	1.35 (1.22–1.48)***
Fair or poor	1.88 (1.68–2.10)***
Current smoker (Ref: Not a current smoker)	1.52 (1.38–1.69)***
Having difficulties paying for medical bills (Ref: Nodifficulties)	2.92 (2.66–3.19)***
Having difficulties paying for medications (Ref: No difficulties)	3.23 (2.88–3.62)***
Income-poverty ratio (Ref: >200 FPL)	
<100 FPL	4.25 (3.75–4.83)***
100–200 FPL	2.97 (2.67–3.30)***
Having health insurance (Ref: No health insurance)	0.88 (0.77–1.00)*
Household size (Ref: 1 member)	
2	0.89 (0.80–0.98)*
3	0.91 (0.80–1.03)
> = 4	0.78 (0.68–0.90)***
Living with a disability (Ref: Living without a disability)	1.55 (1.39–1.73)***
Region (Ref: West)	
Northeast	1.02 (0.89–1.18)
Midwest	0.96 (0.84–1.09)
South	0.92 (0.81–1.04)
Non-metro area (Ref: Metro area)	0.99 (0.87–1.12)
Year (Ref: 2019)	
2020	0.99 (0.90–1.10)
2021	0.74 (0.66–0.82)***

Boldface indicates statistical significance: *, **, *** significant at 5%, 1%, and 0.1% levels, respectively. General Educational Development (GED), Supplemental Nutrition Assistance Program (SNAP), Federal Poverty Level (FPL).

### 3.2. Survey year stratification

The multivariable model stratified by year in Table 3 indicates that bisexual adults reported a significantly higher rate of FI compared to heterosexual adults only during the pandemic; aOR 1.58 (95% CI 1.07–2.33) in 2020 and aOR 1.93 (95% CI 1.37–2.71) in 2021. However, gay/lesbian and other non-heterosexual individuals did not report a significantly different FI rate compared to heterosexual individuals before and during the pandemic. Please see Appendix C for the full table including other independent variables.

**Table 3:** Food insecurity and its association with sexual orientation stratified by survey year.

Variable	2019	2020	2021
Sexual orientation (Ref: Heterosexual)			
Gay/Lesbian	0.89 (0.57–1.42)	1.15 (0.72–1.84)	1.12 (0.66–1.89)
Bisexual	1.30 (0.93–1.81)	1.58 (1.07–2.33)*	1.93 (1.37–2.71)***
Other Non-heterosexual	2.16 (0.99–4.74)	1.07 (0.43–2.65)	1.57 (0.78–3.15)

Results are adjusted for all the independent variables included in Table 1. Numbers reported in the table are adjusted odds ratios with their 95% confidence intervals. Boldface indicates statistical significance: *, **, *** significant at 5%, 1%, and 0.1% levels, respectively.

### 3.3. Race/ethnicity stratification

The multivariable model stratified by race/ethnicity in Table 4 indicates that bisexuals reported a significantly higher rate of FI compared to their heterosexual counterparts only when they identified as non-Hispanic white and non-Hispanic black; aOR 1.58 (95% CI 1.20–2.07) and aOR 2.27 (95% CI 1.21–4.23), respectively. Other non-heterosexual individuals reported a significantly higher rate of FI compared to heterosexuals only when they identified as non-Hispanic black: aOR 3.30 (95% CI 1.24–8.77) (see Appendix C).

**Table 4:** Food insecurity and its association with sexual orientation stratified by race/ethnicity.

Variable	Non-Hispanic white	Non-Hispanic black	Hispanic	Non-Hispanic other
Sexual orientation (Ref: Heterosexual)				
Gay/Lesbian	1.13 (0.76–1.68)	1.07 (0.61–1.89)	0.75 (0.38–1.46)	1.39 (0.45–4.31)
Bisexuals	1.58 (1.20–2.07)**	2.27 (1.21–4.23)*	1.23 (0.77–1.95)	1.04 (0.54–2.00)
Other Non-heterosexual	1.51 (0.86–2.66)	3.30 (1.24–8.77)*	0.51 (0.15–1.71)	1.71 (0.34–8.55)

Results are adjusted for all the independent variables included in Table 1. Numbers reported in the table are adjusted odds ratios with their 95% confidence intervals. Boldface indicates statistical significance: *, **, *** significant at 5%, 1%, and 0.1% levels, respectively.

## 4. Discussion

Prior research demonstrated that sexual minorities experience higher rates of FI compared to heterosexual peers (2–8). FI is linked to several adverse health conditions such as hypertension, obesity, and cardiovascular diseases, which are also disproportionately experienced by sexual minorities (2), suggesting that reducing FI among sexual minorities could contribute significantly to improved health outcomes in the U.S. During the COVID-19 pandemic, discrimination and structural racism, which are known risk factors for FI (4, 7–9), were perpetuated against sexual and racial/ethnic minorities (15–18). However, and to the best of our knowledge, research on FI experiences among sexual minorities before and during the pandemic is limited. While the Household Pulse Survey has been estimating food insufficiency among sexual minorities during the pandemic (23), food insufficiency is a simpler measure of FI, and the Household Pulse Survey does not provide pre-pandemic measures for comparison. In addition, and according to the Household Pulse Survey, people of color who identified as sexual minorities experienced a higher rate of food insufficiency than white heterosexuals, white sexual minorities, and heterosexuals of color (23), which may suggest a need for an intersectional approach that considers membership in multiple historically minoritized groups such as sexual and racial/ethnic minorities (24, 25). Therefore, our goal in this paper was to determine the degree of association between FI and sexual orientation overall, before (2019) and during (2020–2021) the pandemic, and stratified by race/ethnicity.

After adjusting for a variety of independent variables known to be associated with FI, FI disparity remained only between bisexual and heterosexual adults (aOR 1.61 [95% CI 1.31–1.99]). These results suggest that addressing income inequalities and utilization of food resources (e.g., SNAP), while necessary, cannot fully address FI disparities between bisexual and heterosexual adults. To support this claim, in a sub-analysis conducted by authors and not reported here, bisexual individuals reported significantly higher rates of FI compared to heterosexual individuals regardless of their SNAP utilization and across all levels of income-poverty ratios (i.e., less than 100% federal poverty level [FPL], between 100 and 200% FPL, and above 200% FPL). Even after restricting our population sample to those with an income below 200% FPL, still only bisexual individuals reported a higher FI rate compared to heterosexual individuals. Although a singular cause is unknown, this finding suggests that other factors not included in our analysis (such as structural racism, stigma, and discrimination) might better explain FI disparities between bisexuals and heterosexuals.

Using pre-pandemic data (2003–2016), previous literature found that gay/lesbian, bisexual, and other non-heterosexuals were more likely to experience FI compared to heterosexuals (4). However, in our analysis stratified by survey year, in 2019, there were no FI disparities between sexual minority and heterosexual individuals. On the other hand, during the pandemic (2021–2021), bisexual individuals reported a significantly higher rate of FI compared to heterosexuals (aOR 1.58 [95% CI 1.07–2.33] in 2020 and aOR 1.93 [95% CI 1.37–2.71] in 2021), while other sexual minorities included in our sample (i.e., gay/lesbian and other non-heterosexuals) experienced a similar rate of FI as heterosexuals. We hypothesize that the recent surges in discrimination and attacks against sexual minorities during the pandemic (15–17) might have increased FI disparities among bisexuals. In fact, bisexual individuals in general experience higher rates of discrimination from heterosexual individuals compared to other subgroups of sexual minorities (20, 21). Further, bisexual individuals report that psychosocial support services and communities that traditionally target gay or lesbian individuals lead to feelings of further exclusion and social isolation (32). Bisexual individuals are even targeted by other sexual minorities (4, 20–22), a phenomenon often referred to as “double discrimination” (20, 33). Thus, the dual increase in discrimination and decrease in available and inclusive psychosocial services may predispose bisexual individuals to poorer outcomes than other sexual minorities. Our results may suggest a need to continuously monitor FI experiences among sexual minorities and to obtain subgroup data on sexual orientation (i.e., gay/lesbian, bisexuals, other non-heterosexuals), as utilizing a dichotomous variable (sexual minority vs. heterosexual) would have not revealed the FI disparities among bisexuals (3, 6, 7).

Using an intersectionality framework (24, 25), we examined the experiences of FI among people with multiple historically minoritized identities—sexual and racial/ethnic minorities. We found that bisexual individuals reported a significantly higher rate of FI compared to their heterosexual counterparts only when they identified as non-Hispanic white and non-Hispanic black. Other non-heterosexual individuals reported a significantly higher rate of FI compared to heterosexual individuals only when they identified as non-Hispanic black. Prior literature has elucidated the higher burden of stigma on sexual minorities who identify as Black as well (25). While connection to the sexual minority communities can alleviate the burden of stress and stigma among sexual minorities (25), this buffering effect could only be true for white sexual minorities who are not targeted by double discrimination. These results again indicate the importance of simultaneously collecting subgroup data on sexual orientation and race/ethnicity, as disparities in FI within bisexuals were found when stratifying by race and ethnicity.

Considering the dynamic changes in policies endangering or supporting the rights of sexual minorities, FI experiences among sexual minorities should be continuously monitored. For example, in 2022, more than 300 bills were introduced, which targeted the rights of sexual and gender minorities (34, 35). While the majority of these bills target transgender individuals, the mental health of other sexual minority groups has also been affected due to the hostile political climate toward sexual and gender minorities (34). In addition, in many states sexual minorities are not fully protected from discrimination (10, 34, 35). Discrimination can lead to limited access to employment, housing, and social support, which could increase FI rates (4). At the same time, anti-discrimination proposals initiated by the Biden-Harris administration (36) might be a silver lining for sexual minorities. Similar policies to address upstream factors including structural racism, stigma, and discrimination might yield higher improvements in food security among bisexual individuals. Also, this paper is a case study of the possible impact of discrimination on a health-related social risk factor—FI. As FI co-exists with many other social risk factors such as housing and employment insecurity (37), it is worth investigating the extent of other social risk factors among sexual minorities before and after the pandemic.

### 4.1. Limitations

Our results should be interpreted in the light of several limitations. NHIS measures sexual orientation by sexual identity only and does not consider respondents’ sexual attraction and behavior (28). NHIS does not pose the sexual orientation question to children, thus our results might not be generalizable to adolescents. NHIS also does not assess critical factors that are associated with FI including discrimination experiences, language barriers, eating disorders, and utilization of non-federal nutrition assistance programs such as food pantries. Moreover, NHIS’s estimate on unemployment (whether participants did not work in the last week or usually work 35+ hours per week) differs in 2021 from 2019 and 2020 by considering seasonal or contractor workers, hence we did not include it in our analysis. Finally, our results are based on cross-sectional analysis and thus we cannot comment on the causalities between independent variables and FI.

## 5. Conclusion

This cross-sectional study investigated the associations between FI and sexual minority adults. Among sexual minorities in our population sample, bisexual individuals were 61% more likely to experience FI than heterosexual individuals while gay/lesbian and other non-heterosexual individuals experienced similar rates of FI compared to heterosexual individuals. In addition, we found that bisexual individuals who identified as non-Hispanic black were 2.27 times more likely to experience FI compared to heterosexual individuals. The inequities in FI experiences among bisexual and racial/ethnic minority individuals have important implications for public health policy. While individual-level interventions to address FI among sexual and racial/ethnic minorities are warranted, future research should examine everyday experiences of (double) discrimination and lifetime discrimination among these individuals. Intersectional research regarding FI among bisexual and racial/ethnic minority groups would further elucidate how membership in multiple minority groups may contribute to a higher risk of FI. With the growing number of legislative bills targeting the rights of sexual and gender minorities and restrictions on how racism is taught in schools, as new data becomes available, we expect to see a higher burden of FI among bisexual individuals, particularly, bisexual people of color. In order to improve the health of sexual minorities and boost population health, there is a critical need to address FI among these individuals.

## Data availability statement

The original contributions presented in the study are included in the article/Supplementary material, further inquiries can be directed to the corresponding author.

## Author contributions

NS, SB, and FW contributed to the conceptualization, investigation, and methodology of the study, and wrote the original draft of the manuscript. NS compiled the data and conducted the analysis, developed the visualizations, and managed the project. RH, AW, and HS contributed to the conceptualization of the study and reviewed/edited different sections of the manuscript. EG and SJ reviewed/edited different sections of the manuscript. All authors contributed to the manuscript writing and approved the submitted version.

## Conflict of interest

The authors declare that the research was conducted in the absence of any commercial or financial relationships that could be construed as a potential conflict of interest.

## Publisher’s note

All claims expressed in this article are solely those of the authors and do not necessarily represent those of their affiliated organizations, or those of the publisher, the editors and the reviewers. Any product that may be evaluated in this article, or claim that may be made by its manufacturer, is not guaranteed or endorsed by the publisher.
