# Supplementary material for: Disparities in food insecurity between sexual minority and heterosexual adults – a higher burden on bisexual individuals
Source: Front Public Health. 2023 Aug 7;11:1237091. doi: 10.3389/fpubh.2023.1237091 (PMC10441544; doi:10.3389/fpubh.2023.1237091)
Supplement: Supplementary file 1 [file Table_1.docx]

Supplementary Material

Disparities in Food Insecurity between Sexual Minority and Heterosexual Adults – A Higher Burden on Bisexual Individuals

Nasser Sharareh, Ph.D.,^1*^ Sara Bybee, LCSW, Ph.D.,^2^ Evan Goldstein, Ph.D.,^1^ Shannon Jones, M.S.,^3^ Rachel Hess, MD, MS,^1, †^ Andrea Wallace, Ph.D., RN, FAAN,^1, 2, †^ Hilary Seligman, MD, MAS,^4, †^ Fernando A. Wilson, Ph.D.^1, 5, 6^

^1^Department of Population Health Sciences, Spence Fox Eccles School of Medicine, University of Utah, Salt Lake City, UT, United States

^2^College of Nursing, University of Utah, Salt Lake City, UT, United States

^3^Department of Nutrition and Integrative Physiology, University of Utah, Salt Lake City, UT, United States

^4^Department of Medicine, School of Medicine, University of California, San Francisco, San Francisco, CA, United States

^5^Matheson Center for Health Care Studies, University of Utah, Salt Lake City, UT, United States

^6^Department of Economics, College of Social and Behavioral Science, University of Utah, Salt Lake City, UT, United States

*** Correspondence:**Dr. Nasser Sharareh
[nasser.sharareh@hsc.utah.edu](mailto:nasser.sharareh@hsc.utah.edu)

# Supplementary Data

## Appendix A - Variables

All the variables that were used for our analysis, their names as appeared on the NHIS data, and the questions that were asked for each variable are reported here. Notice that some variables have different names in 2021 compared to 2019 and 2020. We also reclassification most of the variables using R software; the codes are reported in **Appendix B.**

**Dependent Variable**

Food insecurity (FDSCAT3_A): The food security status of the household was determined by responses to 10 food security questions (FDSRUNOUT_C/FDSRUNOUT_C through FDSNEDAYS_A/FDSNEDAYS_C) asked in the adult interview.

USDA’s food security module has 18 items. The food security status of children in the household is assessed by responses to the child-referenced items (items 11-18). However, NHIS only asks the first 10 items from respondents and does not ask the child-referenced questions. Therefore, we only included adults, as we have access to adult-referenced questions (1-10). The only difference is that USDA measures food insecurity over the past 12 months, while NHIS measures food insecurity over the past 30 days. See below the 10 adult-referenced questions.

1. “We worried whether our food would run out before we got money to buy more.” Was that often, sometimes, or never true for you in the last 30 days?
2. “The food that we bought just didn’t last, and we didn’t have money to get more.” Was that often, sometimes, or never true for you in the last 30 days?
3. “We couldn’t afford to eat balanced meals.” Was that often, sometimes, or never true for you in the last 30 days?
4. In the last 30 days, did you or other adults in the household ever cut the size of your meals or skip meals because there wasn’t enough money for food? (Yes/No)
5. (If yes to question 4) In the last 30 days, how often did this happen?
6. In the last 30 days, did you ever eat less than you felt you should because there wasn’t enough money for food? (Yes/No)
7. In the last 30 days, were you ever hungry, but didn’t eat, because there wasn’t enough money for food? (Yes/No)
8. In the last 30 days, did you lose weight because there wasn’t enough money for food? (Yes/No)
9. In the last 30 days did you or other adults in your household ever not eat for a whole day because there wasn’t enough money for food? (Yes/No)
10. (If yes to question 9) In the last 30 days, how many days did this happen?

Questions 1–3 are coded as affirmative (i.e., possibly indicating food insecurity) if the response is “often” or “sometimes.” Questions 5 and 10 are coded as affirmative if the response is more than 5 days. The remaining questions are coded as affirmative if the response is “yes.”

3 or more affirmative responses are considered food insecure.

**Main Independent Variable**

Sexual Orientation (ORIENT_A): Do you think of yourself as gaylesbian; straight, that is, not gay/lesbian and bisexual; something else; or you don't know the answer?

A categorical variable was used to indicate whether a person is heterosexual, gay/lesbian, bisexual, or other non-heterosexual individuals (i.e., something else), which could include identities such as asexual and pansexual.

**Covariates**

NHIS collects data on a variety of factors. Based on the literature and our expertise, we used the following demographic information: immigration status, income, race/ethnicity, marital status, gender, age, employment, education, health insurance, household size, disability status, region, metro/nonmetro area, health status, smoking status, and difficulties paying for medications and medical bills. We also acquired data on SNAP utilization.

Below, is the question that NHIS ask participants for each covariate.

- Born in the US (NATUSBORN_A): were you born in the U.S. or a U.S. territory?
- Citizenship Status (CITZNSTP_A): are you a citizen of U.S.?
- Based on responses to these two questions, participants were categorized as born in the U.S., naturalized citizens, or non-citizens.
- Income (RATCAT_A): ratio of family income to poverty thresholds sample adult’s family
- Race/Ethnicity (HISPALLP_A): Single and multiple race groups with Hispanic origin: Hispanic, Non-Hispanic White, Non-Hispanic Black, and Non-Hispanic other (including AIAN, Asian, other single and multiple races).
- Marital Status (MARITAL_A): Are you now married, living with a partner together as an unmarried couple, or neither?
- Age (AGEP_A): Age of sample adult. Age was categorized into four categories. 18-34; 35-49; 50-64; and +65.
- Sex (Sex_A): Female or Male
- Health Insurance (HICOV_A): Are you covered by any kind of health insurance or some other kind of health care plan?
- Education (MAXEDUC_A or MAXEDUCP_A in 2021): highest level of education of all the adults in SA’s family
- Household Size: number of household members (Sum of PCNT18UPTC and PCNTLT18TC)
- Disability Status (DISAB3_A): The Washington Group Short Set Composite Disability Indicator
- Region (REGION): Northeast, Midwest, South, and West.
- Metro/nonmetro area (URBRRL): 2013 NCHS Urban-Rural Classification Scheme for Counties
- Health Status (PHSTAT_A): Would you say your health in general is excellent, very good, good, fair, or poor?
- Current Smoker (SMKCIGST_A): Current smokers vs former or never smoker.
- Difficulties paying for medical bills (PAYBLL12M_A): In the past 12 months, did you/anyone in the family have problems paying or were unable to pay medical bills?
- Difficulties paying for medications (RXDG12M_A): During the past 12 months, was there any time when you needed prescription medication, but did not get it because of the cost?
- SNAP Utilization (FSNAP12M_A): At any time in the last 12 months did you/any family members living here received food stamp benefits/[state food stamp program name]?

Year: 2019 is 0, 2020 is 1, and 2021 is 2.

## Appendix B – R Codes

We reclassified all the variables from NHIS using R software. For instance, for Food Insecurity, NHIS coded food-secure as 1, low food security as 2, very low food security as 3, and not ascertained as 8. We recoded 1 to 0, 2 and 3 to 1, and 8 to missing (i.e., N/A). Now, food-insecure are identified as 1 and the reference variable is 0, meaning food-secure. The rest of the variables are reclassified similarly.

Lines that start with “#” are for further explanations.

OurData <- OurData %>%

#dependent variable

mutate(FoodInsecurity=recode(FoodInsecurity, '1'=0, '2'=1, '3'=1, '8'=NA_real_))%>%

#main independent variable

#heterosexual is 0, gay/lesbian is 1, bisexual is 2, something else is 3, i don't know + refused + not ascertained + don't know is NA

mutate(SexOrientation=recode(SexOrientation, '1'=1, '2'=0, '3'=1, '4'=1,'5'=1, '7'=NA_real_, '8'=NA_real_, '9'=NA_real_))%>%

#other independent variables

mutate(USBorn=recode(USBorn, '1'=1, '2'=0, '7'=NA_real_, '8'=NA_real_, '9'=NA_real_))%>%

#US Citizens are 0

mutate(USCitizen=recode(USCitizen, '1'=1, '2'=0, '7'=NA_real_, '8'=NA_real_, '9'=NA_real_))%>%

#Straight is 0, sexual minorities is 1,

# more than 200% FPL is 0, less than 100% FPL is 1, between 100% and 200% FPL is 2

mutate(IncomePoverty=recode(IncomePoverty, '1'=1, '2'=1,'3'=1, '4'=2, '5'=2, '6'=2, '7'=2, '8'=0, '9'=0, '10'=0, '11'=0, '12'=0, '13'=0, '14'=0, '98'=NA_real_))%>%

#NH White is 0, NH black is 1, Hispanic is 2, NH others 3

mutate(RaceEthnicity=recode(RaceEthnicity, '1'=2, '2'=0, '3'=1, '4'=3, '5'=3, '6'=3, '7'=3,

'97'=NA_real_, '98'=NA_real_, '99'=NA_real_))%>%

#married is 1

mutate(Married=recode(Married, '1'=1, '2'=0, '3'=0, '7'=NA_real_, '8'=NA_real_, '9'=NA_real_))%>%

#Famele is 1

mutate(Female=recode(Female, '1'=0, '2'=1, '7'=NA_real_, '8'=NA_real_, '9'=NA_real_))%>%

#highest level of education of all the adults in SA's Family

#no diplome is 0, GED and high school 1, some college and associate is 2, bachelor is 3, higher graduare is 4

mutate(Education=recode(Education, '0'=0, '1'=0, '2'=0, '3'=1, '4'=1, '5'=2, '6'=2, '7'=2, '8'=3, '9'=4, '10'=4,

'11'=4, '97'=NA_real_, '98'=NA_real_, '99'=NA_real_))%>%

mutate(Insured=recode(Insured, '1'=1, '2'=0, '7'=NA_real_, '8'=NA_real_, '9'=NA_real_))%>%

#1 is 0, 2 is 1, 3 is 2, more than 4 is 3

mutate(HouseholdSize=recode(HouseholdSize, '1'=0, '2'=1, '3'=2, '4'=3, '5'=3, '6'=3, '16'=NA_real_))%>%

mutate(Disabled=recode(Disabled, '1'=1, '2'=0, '9'=NA_real_))%>%

#metro is 0, non metro is 1

mutate(Metro=recode(Metro, '1'=0, '2'=0, '3'=0, '4'=1))%>%

#West is 0; northeast is 1; midwest is 2; south is 3

mutate(Region=recode(Region, '4'=0, '1'=1, '2'=2, '3'=3))%>%

#excellent and very good is 0, good is 1, fair and poor is 2

mutate(HealthStatus=recode(HealthStatus, '1'=0, '2'=0, '3'=1, '4'=2, '5'=2, '7'=NA_real_, '8'=NA_real_, '9'=NA_real_))%>%

#former smoker and never smoker and not known is 0, current is 1

mutate(CurrentSmoker=recode(CurrentSmoker, '1'=1, '2'=1, '3'=0, '4'=0, '5'=0, '9'=NA_real_))%>%

mutate(SNAP=recode(SNAP, '1'=1, '2'=0, '7'=NA_real_, '8'=NA_real_, '9'=NA_real_))%>%

#2019 is the reference

mutate(Year=recode(Year, '2019'=0, '2020'=1, '2021'=2))

#create a new variable for immigration status. US Born as the reference (0), non citizens as 1, and naturalized citizens as 2

OurData <- OurData %>%

mutate(ImmigrationStatus = ifelse(USBorn==1 & USCitizen==1 , 0,

ifelse(USBorn==0 & USCitizen==0, 1,

ifelse(USBorn==0 & USCitizen==1, 2, NA))))

#create a categorical variable for Age

OurData$AgeCat <- cut(OurData$Age,

breaks = c(17,34,49,64,100),

labels = c('18-34','35-49','50-64','+65'))

#18-34 is 0; 35-49 is 1; 50-64 is 2; +65 is 3

OurData <- OurData %>%

mutate(AgeCat=recode(AgeCat, '18-34'=0, '35-49'=1, '50-64'=2, '+65'=3))

## Appendix C – Tables

Table 1: Food insecurity and its association with sexual orientation stratified by survey year (Full version of Table 3 in the Manuscript)

| **Variable** | **2019 (n = 30,115)** | **2020 (n = 29,533)** | **2021 (n = 27,454)** |
| --- | --- | --- | --- |
| Sexual Orientation (Ref: Heterosexuals) |  |  |  |
| Gay/Lesbian | 0.89 (0.57-1.42) | 1.15 (0.72-1.84) | 1.12 (0.66-1.89) |
| Bisexuals | 1.30 (0.93-1.81) | **1.58 (1.07-2.33)*** | **1.93 (1.37-2.71)***** |
| Other Non-Heterosexuals | 2.16 (0.99-4.74) | 1.07 (0.43-2.65) | 1.57 (0.78-3.15) |
| Immigration Status (Ref: U.S. Born) |  |  |  |
| Non-Citizens | 1.11 (0.87-1.42) | **1.56 (1.17**-**2.1)**** | **1.58 (1.16**-**2.14)**** |
| Naturalized Citizens | 1.00 (0.78-1.27) | 1.26 (0.96-1.64) | **1.47 (1.12**-**1.95)**** |
| Race/Ethnicity (Ref: Non-Hispanic White) |  |  |  |
| Non-Hispanic Black | **1.73 (1.46**-**2.06)***** | **1.68 (1.36, 2.06)***** | **2.00 (1.62**-**2.47)***** |
| Hispanic | 1.53 (1.25-1.87)*** | **1.29 (1.01**-**1.64)*** | 1.05 (0.83-1.32) |
| Non-Hispanic Other | **1.56 (1.15**-**2.11)**** | **1.43 (1.10**-**1.87)**** | 1.30 (0.82-2.06) |
| Married (Ref: Not Married) | **0.78 (0.67**-**0.90)***** | **0.79 (0.66**-**0.95)*** | 0.91 (0.76-1.08) |
| Female (Ref: Male) | 1.09 (0.96-1.24) | 1.10 (0.96-1.26) | 0.99 (0.85-1.15) |
| Age (Ref: 18-34) |  |  |  |
| 35-49 | 1.15 (0.97-1.35) | 0.98 (0.78-1.22) | 0.96 (0.79-1.16) |
| 50-64 | 0.90 (0.75-1.09) | 0.85 (0.68-1.06) | **0.76 (0.60**-**0.94)*** |
| 65+ | **0.54 (0.44**-**0.67)***** | **0.50 (0.39**-**0.64)***** | **0.42 (0.32**-**0.55)***** |
| Education (Ref: No Diploma) |  |  |  |
| GED or High School | 0.97 (0.78-1.20) | 1.00 (0.78-1.28) | 0.78 (0.60-1.02) |
| Some College | 0.91 (0.74-1.12) | 1.06 (0.82-1.38) | 0.80 (0.61-1.03) |
| Bachelor | **0.54 (0.41**-**0.70)***** | **0.58 (0.42**-**0.80)**** | **0.61 (0.44**-**0.84)**** |
| Higher Graduate | **0.38 (0.27**-**0.54)***** | **0.43 (0.30**-**0.61)***** | **0.40 (0.27**-**0.58)***** |
| SNAP Utilizer (Ref: Not Using SNAP) | **2.49 (2.13**-**2.92)***** | **2.09 (1.72**-**2.53)***** | **1.79 (1.47**-**2.18)***** |
| Self-Reported Health Status (Ref: Excellent or Very Good) |  |  |  |
| Good | **1.27 (1.08**-**1.48)**** | **1.39 (1.17**-**1.66)***** | **1.36 (1.13**-**1.64)**** |
| Fair or Poor | **1.77 (1.48**-**2.10)***** | **1.94 (1.59**-**2.38)***** | **1.93 (1.57**-**2.38)***** |
| Current Smoker (Ref: Not a Current Smoker) | **1.66 (1.43**-**1.93)***** | **1.38 (1.14**-**1.66)***** | **1.52 (1.25**-**1.86)***** |
| Having Difficulties Paying for Medical Bills (Ref: No Difficulties) | **2.92 (2.52**-**3.37)***** | **2.86 (2.43**-**3.36)***** | **3.00 (2.52**-**3.57)***** |
| Having Difficulties Paying for Medications (Ref: No Difficulties) | **3.29 (2.76**-**3.91)***** | **3.21 (2.62**-**3.95)***** | **3.24 (2.56**-**4.10)***** |
| Income-Poverty ratio (Ref: > 200 FPL) |  |  |  |
| < 100 FPL | **3.35 (2.76**-**4.07)***** | **5.30 (4.19**-**6.70)***** | **4.51 (3.59**-**5.67)***** |
| 100-200 FPL | **2.41 (2.05**-**2.85)***** | **3.61 (2.98**-**4.38)***** | **3.16 (2.62**-**3.81)***** |
| Having Health Insurance (Ref: No Health Insurance) | 1.00 (0.81-1.22) | 0.82 (0.65-1.04) | 0.81 (0.64-1.04) |
| Household Size (Ref: 1 member) |  |  |  |
| 2 | 0.95 (0.81-1.10) | 0.84 (0.69-1.01) | 0.87 (0.72-1.06) |
| 3 | 0.84 (0.69-1.02) | 0.98 (0.78-1.25) | 0.93 (0.75-1.14) |
| >=4 | 0.82 (0.65-1.04) | 0.89 (0.69-1.14) | 0.60 (0.46-0.78)*** |
| Disabled** (Ref: Not Disabled) | **1.56 (1.30**-**1.87)***** | **1.51 (1.25**-**1.83)***** | **1.56 (1.28**-**1.90)***** |
| Region (Ref: West) |  |  |  |
| Northeast | 1.00 (0.80-1.26) | **1.30 (1.00**-**1.69)*** | 0.78 (0.60-1.00) |
| Midwest | 1.00 (0.82-1.22) | 1.08 (0.85-1.37) | 0.82 (0.65-1.05) |
| South | 0.93 (0.77-1.13) | 1.06 (0.85-1.33) | **0.78 (0.63**-**0.98)*** |
| Non-Metro Area (Ref: Metro Area) | 0.95 (0.78-1.15) | 0.92 (0.74-1.14) | 1.13 (0.87-1.47) |

Boldface indicates statistical significance: *, **, *** significant at 5%, 1%, and 0.1% levels, respectively.

Abbreviations: General Educational Development (GED), Supplemental Nutrition Assistance Program (SNAP), Federal Poverty Level (FPL).

Table 2: Food insecurity and its association with sexual orientation stratified by race/ethnicity (Full version of Table 3 in the Manuscript)

| **Variable** | **Non-Hispanic White (n=60,279)** | **Non-Hispanic Black (8,926)** | **Hispanic (n=11,056)** | **Non-Hispanic Other (n=6,841)** |
| --- | --- | --- | --- | --- |
| Sexual Orientation (Ref: Heterosexuals) |  |  |  |  |
| Gay/Lesbian | 1.13 (0.76-1.68) | 1.07 (0.61-1.89) | 0.75 (0.38-1.46) | 1.39 (0.45-4.31) |
| Bisexuals | **1.58 (1.20-2.07)**** | **2.27 (1.21-4.23)*** | 1.23 (0.77-1.95) | 1.04 (0.54-2.00) |
| Other Non-Heterosexuals | 1.51 (0.86-2.66) | **3.30 (1.24-8.77)*** | 0.51 (0.15-1.71) | 1.71 (0.34-8.55) |
| Immigration Status (Ref: U.S. Born) |  |  |  |  |
| Non-Citizens | 0.94 (0.55-1.61) | **2.64 (1.74-4.00)***** | **1.35 (1.09-1.69)**** | 0.97 (0.62-1.54) |
| Naturalized Citizens | 0.91 (0.68-1.23) | **1.59 (1.18-2.15)**** | 1.00 (0.78-1.28) | 1.03 (0.71-1.50) |
| Married | **0.76 (0.65-0.87)***** | 0.92 (0.73-1.15) | 0.86 (0.72-1.03) | 0.91 (0.64-1.28) |
| Female | 1.07 (0.95-1.20) | 1.13 (0.96-1.34) | 1.05 (0.88-1.25) | 1.09 (0.83-1.44) |
| Age (Ref: 18-34) |  |  |  |  |
| 35-49 | 1.02 (0.87-1.20) | 1.02 (0.80-1.29) | 0.98 (0.78-1.24) | 1.36 (0.95-1.95) |
| 50-64 | **0.71 (0.60-0.84)***** | 0.82 (0.64-1.04) | 0.96 (0.74-1.24) | **1.66 (1.08-2.55)*** |
| 65+ | **0.35 (0.29-0.42)***** | **0.61 (0.45-0.82)**** | **0.70 (0.51-0.95)*** | 1.03 (0.61-1.74) |
| Education (Ref: No Diploma) |  |  |  |  |
| GED or High School | **0.77 (0.62-0.96)*** | 0.99 (0.76-1.28) | 0.96 (0.75-1.23) | 1.42 (0.89-2.28) |
| Some College | 0.92 (0.74-1.15) | 0.80 (0.60-1.05) | 0.87 (0.67-1.13) | 1.17 (0.73-1.90) |
| Bachelor | **0.54 (0.41-0.70)***** | **0.64 (0.44-0.94)*** | **0.62 (0.45-0.86)**** | **0.54 (0.31-0.95)*** |
| Higher Graduate | **0.41 (0.30-0.54)***** | **0.49 (0.32-0.74)***** | **0.39 (0.24-0.62)***** | **0.34 (0.16-0.70)**** |
| SNAP | **2.52 (2.15-2.95)***** | **1.68 (1.36-2.07)***** | **1.80 (1.46-2.22)***** | **2.76 (1.99-3.81)***** |
| Self-Reported Health Status (Ref: Excellent or Very Good) |  |  |  |  |
| Good | **1.66 (1.45-1.91)***** | 1.11 (0.89-1.38) | 1.07 (0.86-1.32) | 1.29 (0.92-1.80) |
| Fair or Poor | **2.12 (1.81-2.48)***** | **1.60 (1.26-2.04)***** | **1.65 (1.30-2.09)***** | **1.94 (1.33-2.82)***** |
| Current Smoker | **1.50 (1.32-1.70)***** | **1.55 (1.25-1.92)***** | **1.67 (1.30-2.15)***** | 1.05 (0.70-1.59) |
| Difficulties Paying for Medical Bills | **3.13 (2.75-3.56)***** | **2.54 (2.09-3.09)***** | **2.56 (2.10-3.13)***** | **3.32 (2.31-4.78)***** |
| Difficulties Paying for Medications | **3.11 (2.67-3.63)***** | **2.94 (2.32-3.71)***** | **3.54 (2.76-4.53)***** | **4.64 (2.81-7.67)***** |
| Income-Poverty ratio (Ref: > 200 FPL) |  |  |  |  |
| < 100 FPL | **4.97 (4.18-5.90)***** | **2.92 (2.20-3.88)***** | **3.84 (2.95-5.01)***** | **3.91 (2.57-5.94)***** |
| 100-200 FPL | **3.58 (3.11-4.14)***** | **2.48 (1.96-3.15)***** | **2.19 (1.76-2.72)***** | **3.04 (2.08-4.43)***** |
| Having Health Insurance | **0.79 (0.64-0.96)*** | 1.06 (0.80-1.41) | 0.95 (0.75-1.19) | 0.64 (0.39-1.04) |
| Household Size (Ref: 1 member) |  |  |  |  |
| 2 | 0.89 (0.77-1.03) | **0.75 (0.61-0.93)**** | 1.04 (0.81-1.33) | 1.23 (0.85-1.78) |
| 3 | 0.90 (0.75-1.08) | 0.85 (0.66-1.09) | 1.04 (0.80-1.36) | 0.93 (0.64-1.37) |
| >=4 | **0.69 (0.56-0.86)***** | **0.70 (0.53-0.93)*** | **0.94 (0.70-1.25)***** | 1.15 (0.72-1.85) |
| Disabled** | **1.67 (1.43-1.94)***** | 1.30 (1.00-1.70) | **1.59 (1.24-2.03)***** | 1.22 (0.77-1.92) |
| Region (Ref: West) |  |  |  |  |
| Northeast | 1.03 (0.85-1.25) | 1.25 (0.85-1.84) | 1.15 (0.86-1.53) | 0.95 (0.64-1.42) |
| Midwest | 0.96 (0.81-1.14) | **1.49 (1.03-2.16)*** | 0.95 (0.71-1.27) | 0.69 (0.45-1.04) |
| South | 0.95 (0.81-1.13) | 1.27 (0.91-1.78) | 1.01 (0.82-1.25) | **0.56 (0.39-0.82)**** |
| Non-Metro Area | 0.96 (0.83-1.12) | 0.95 (0.71-1.27) | **0.60 (0.42-0.87)***** | **1.68 (1.10-2.55)*** |
| Year (Ref: 2019) |  |  |  |  |
| 2020 | 0.93 (0.81-1.07) | 1.00 (0.82-1.23) | 1.10 (0.88-1.36) | 0.98 (0.71-1.35) |
| 2021 | **0.74 (0.64-0.85)***** | **0.79 (0.64-0.97)*** | **0.65 (0.53-0.80)***** | 0.71 (0.48-1.04) |

Boldface indicates statistical significance: *, **, *** significant at 5%, 1%, and 0.1% levels, respectively.

Abbreviations: General Educational Development (GED), Supplemental Nutrition Assistance Program (SNAP), Federal Poverty Level (FPL).

**
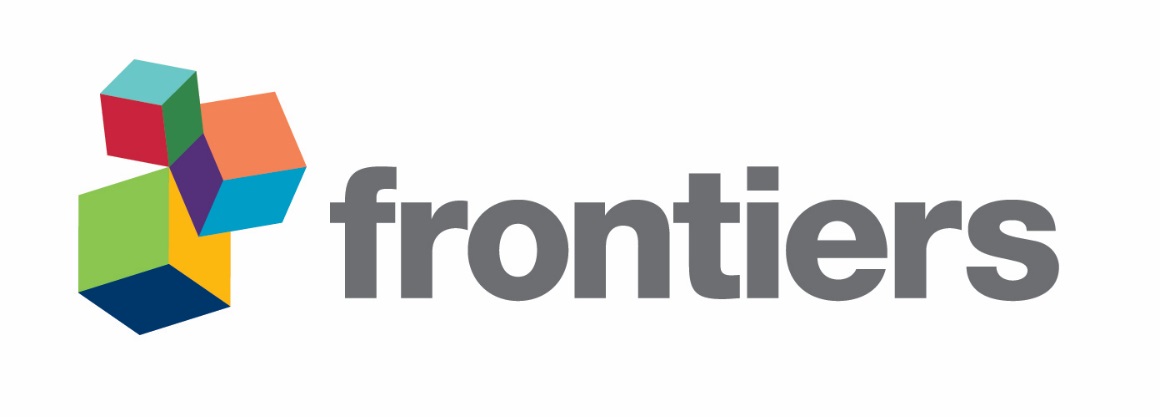
**
